# Factors Engaging Users of Diabetes Social Media Channels on Facebook, Twitter, and Instagram: Observational Study

**DOI:** 10.2196/21204

**Published:** 2020-09-29

**Authors:** Elia Gabarron, Dillys Larbi, Enrique Dorronzoro, Per Erlend Hasvold, Rolf Wynn, Eirik Årsand

**Affiliations:** 1 Norwegian Centre for E-health Research University Hospital of North Norway Tromsø Norway; 2 Department of Electronic Technology University of Seville Seville Spain; 3 Medical Division University Hospital of North Norway Tromsø Norway; 4 Department of Clinical Medicine UiT The Arctic University of Norway Tromsø Norway; 5 Department of Computer Science UiT The Arctic University of Norway Tromsø Norway

**Keywords:** social media, Facebook, Twitter, Instagram, diabetes, engagement

## Abstract

**Background:**

Diabetes patient associations and diabetes-specific patient groups around the world are present on social media. Although active participation and engagement in these diabetes social media groups has been mostly linked to positive effects, very little is known about the content that is shared on these channels or the post features that engage their users the most.

**Objective:**

The objective of this study was to analyze (1) the content and features of posts shared over a 3-year period on 3 diabetes social media channels (Facebook, Twitter, and Instagram) of a diabetes association, and (2) users’ engagement with these posts (likes, comments, and shares).

**Methods:**

All social media posts published from the Norwegian Diabetes Association between January 1, 2017, and December 31, 2019, were extracted. Two independent reviewers classified the posts into 7 categories based on their content. The interrater reliability was calculated using Cohen kappa. Regression analyses were carried out to analyze the effects of content topic, social media channel, and post features on users’ engagement (likes, comments, and shares).

**Results:**

A total of 1449 messages were posted. Posts of interviews and personal stories received 111% more likes, 106% more comments, and 112% more shares than miscellaneous posts (all *P<*.001). Messages posted about awareness days and other celebrations were 41% more likely to receive likes than miscellaneous posts (*P<*.001). Conversely, posts on research and innovation received 31% less likes (*P<*.001), 35% less comments (*P=*.02), and 25% less shares (*P=*.03) than miscellaneous posts. Health education posts received 38% less comments (*P=*.003) but were shared 39% more than miscellaneous posts (*P=*.007). With regard to social media channel, Facebook and Instagram posts were both 35 times more likely than Twitter posts to receive likes, and 60 times and almost 10 times more likely to receive comments, respectively (*P<*.001). Compared to text-only posts, those with videos had 3 times greater chance of receiving likes, almost 4 times greater chance of receiving comments, and 2.5 times greater chance of being shared (all *P<*.001). Including both videos and emoji in posts increased the chances of receiving likes by almost 7 times (*P<*.001). Adding an emoji to posts increased their chances of receiving likes and being shared by 71% and 144%, respectively (*P<*.001).

**Conclusions:**

Diabetes social media users seem to be least engaged in posts with content topics that a priori could be linked to greater empowerment: research and innovation on diabetes, and health education. Diabetes social media groups, public health authorities, and other stakeholders interested in sharing research and innovation content and promoting health education on social media should consider including videos and emoji in their posts, and publish on popular and visual-based social media channels, such as Facebook and Instagram, to increase user engagement.

**International Registered Report Identifier (IRRID):**

RR2-10.1186/s12913-018-3178-7

## Introduction

Patient associations and patient groups from around the world are increasingly more present on social media. Being both ubiquitous and freely accessible, social media channels allow patient associations to share content and connect with individuals interested in their health condition. Representing one of the most prevalent chronic diseases worldwide, diabetes associations and diabetes patient groups can also be found on social media [1-4].

Belonging to health-related groups on social media has been linked to several benefits for users, including a reduction in feelings of isolation [5,6], an increased sense of belonging [5,7], positive confirmation of their own situation [5,8], an enhanced sense of well-being [9,10], increased feelings of empowerment [2,11-15], and better health outcomes for users of diabetes-specific social media [11,16-19].

However, although active participation and engagement in diabetes-specific social media groups is mostly linked to positive effects, very little is known about the content that is shared on these channels or what features of posts engage their users the most. In a previous study [20], we surveyed followers of the Norwegian Diabetes Association’s social media channels, and we found that almost all the respondents wanted more content about research and innovation on diabetes in social media groups, preferably in text format. However, other previous studies have reported that social media groups for patients with diabetes mostly shared content about diabetes self-management [1-4], scientific content [3,4], health care services [3,4], diabetes awareness [3,4], personal stories [2], or humor [2].

One way of assessing if posted messages engage users of diabetes-related social media is by measuring the posts’ received feedback in the form of likes, shares, and comments. Likes and shares are a form of communication that allows social media users to provide feedback to other users with a simple click [21,22]. This quick interaction (ie, likes and shares) signals the user’s agreement with the published content [21], and is perceived as a way of supporting the post [22]. Writing comments on social media, which requires more effort than a simple click, has been associated with either strong agreement or strong disagreement with the post [21,23]. Previous studies have reported that Facebook posts including media (ie, pictures, videos, or emoji), providing links, or expressing positive sentiments engage users the most [24], while posts including links and expressing negative sentiments are the least shared [25]. Social media posts dealing with diabetes management and expressing negative sentiments seem to receive more likes when the post is text-only, and less likes when the post includes images [25].

The objective of this study was to analyze the content topic and features of posts that were shared over 3 years in the 3 diabetes-related social media channels (Facebook, Twitter, and Instagram) of the Norwegian Diabetes Association, as well as the users’ engagement with these posts. This study is part of a participatory research project on the use of social media for health promotion in diabetes [26]. This project is carried out in collaboration with Diabetesforbundet, the main diabetes association in Norway [27]. By January 2020, the association had more than 34,000 followers on Facebook, more than 7000 followers on Instagram, and more than 3000 followers on Twitter.

## Methods

### Data Extraction

All social media posts from the Norwegian Diabetes Association (on Facebook, Twitter, and Instagram) published between January 1, 2017, and December 31, 2019, were extracted and included in the study (no posts were excluded or removed from the analysis). The social media posts were extracted using the manager tool for Facebook, manually for Instagram, and using the standard application programming interface (API) for Twitter. Using the appropriate functions, the standard API allows us to gather all tweets from a specific timeframe. In this way, a PHP script was programmed to query Twitter for all the tweets made by the Norwegian Diabetes Association during the study’s timeframe. The Twitter data, including the text and the tweet metadata (ie, date of publishing, likes, retweets, etc), were then exported into a Microsoft Excel file document. The following information was extracted from each post: text message. post features (ie, use of emoji, picture, and/or video), and number of likes, comments, and shares. For Facebook, we collected the total number of likes, including the reactions, for each post. For Instagram, we only extracted likes and comments because shares were not an available option at the time of the study.

### Code Categories

We classified the content topics into 7 categories. These categories were based on findings from our previous studies [3,4,20], and consisted of (1) health education (including self-management and self-monitoring, information about the condition, and promotion of exercise), (2) research and innovation on diabetes (where results of an investigation were reported), (3) diabetes-related technology (including information about apps, blood-glucose monitors, and insulin pumps, but unrelated to politics), (4) interviews and personal stories, (5) awareness days and other celebrations, (6) recipes and food-related information, and (7) miscellaneous (including information about politics related to diabetes, announcements of conferences, courses, meetings, and events).

Two independent reviewers classified the text message of each post according to its main topic. When a post was considered to fall into more than one category, reviewers were trained and instructed to choose the main topic among the 7 possible options. Discrepancies in the posts’ classification were discussed with a third reviewer until reaching consensus. The inter-rater reliability was calculated using Cohen kappa analysis.

### Statistical Analyses

All descriptive and regression analyses were performed using SPSS software (version 25; IBM Corp). The dependent variables in the regression analyses were the number of likes, comments, and shares, which were count data and non-normally distributed. Negative binomial regression models emerged as most appropriate based on the overdispersion parameters and the goodness-of-fit indices. For each of the dependent variables, we performed multilevel negative binomial regression, with the predictors being the independent nominal variables. The largest category in each group was used as the reference: content topic (reference group: miscellaneous), social media channel (reference group: Twitter), and post features (reference group: text only). We determined the interaction between the content topic, social media channel, and post features. The level of significance was set at *P<*.05.

### Ethics

The study protocol was exempted from requiring ethical approval by the Norwegian Regional Ethics Committee (2017/764/REK Sør-ØstC), as it falls outside the scope of the Norwegian Health Research Act. The treatment of personal information was approved by the Data Protection Officer at the University Hospital of North Norway (ref 0720).

## Results

### Sample

During the 3-year period of the study, the Norwegian Diabetes Association posted a total of 1449 messages on their social media channels: 718 (49.55%) were posted on Twitter, 530 (36.58%) were posted on Facebook, and 201 (13.87%) were posted on Instagram. The number of posts on each social media channel according to post features (text only, inclusion of picture, video, and/or emoji) is summarized in Table 1.

**Table 1:** Number of posts on 3 social media channels according to post features.

		Without emoji	With emoji	Total
**Facebook**		410	120	530
	Text post	247	73	320
	Text + picture	105	30	135
	Text + video	58	17	75
**Twitter**		698	20	718
	Text post	455	8	463
	Text + picture	225	11	236
	Text + video	18	1	19
**Instagram**		15	186	201
	Text post	N/A^a^	N/A	N/A
	Text + picture	12	169	181
	Text + video	3	17	20
Total		1123	326	1449

^a^N/A: not applicable

### Content Topic Classification

**Figure 1:**
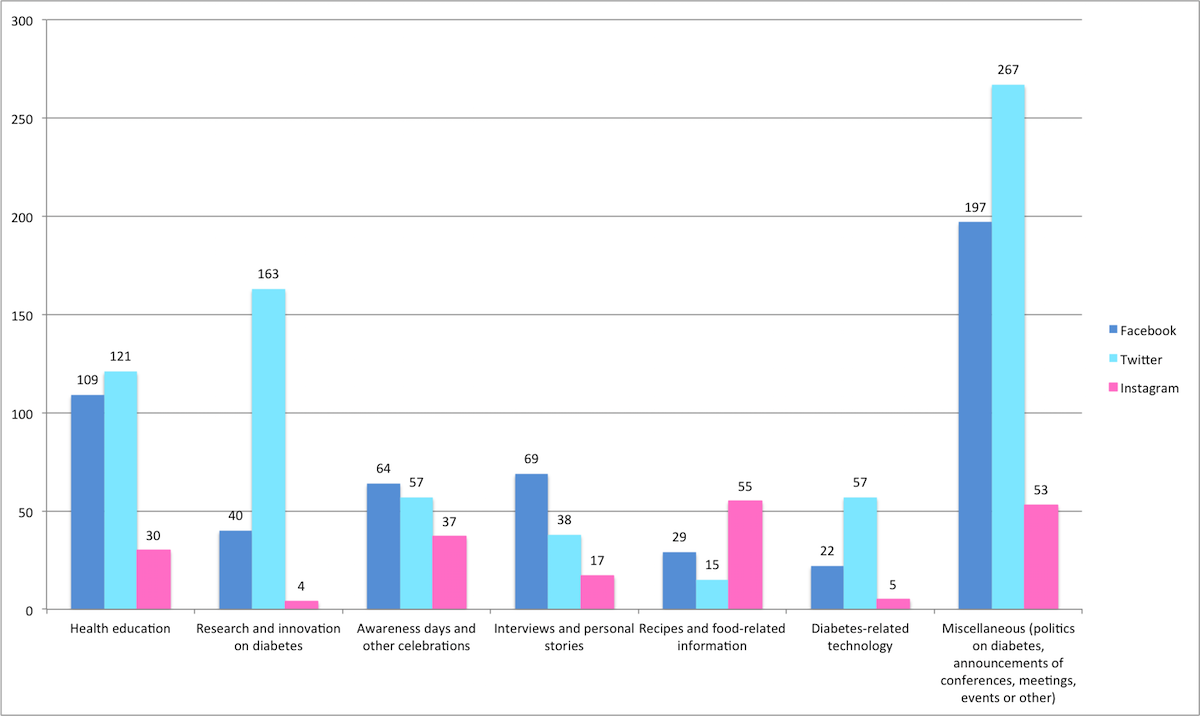
Number of diabetes-related posts on 3 social media channels according to content (January 1, 2017, to December 31, 2019).

The interrater agreement of the posts’ main topic was found to be substantial (κ=0.695, κ=0.780, and κ=0.789, for Twitter, Facebook, and Instagram posts, respectively) [28]. Most of the social media posts fell into the miscellaneous category (517/1449, 35.68%), followed by health education (260/1449, 17.94%), and research and innovation on diabetes (207/1449, 14.29%). With only 84 posts in the 3-year period, diabetes-related technology was the least represented category (5.80%). The total number of posts on each social media channel according to content is shown in Figure 1.

### Engagement: Likes, Comments, and Shares

The effect of content topic, social media channel, and post features on the measures of users’ engagement was analyzed using negative binomial regression. 

The regression analysis showed that posts of interviews and personal stories received 111% more likes, 106% more comments, and 112% more shares than miscellaneous posts (*P<*.001 for all). Posts on the topics of awareness days and other celebrations were 41% more likely to receive likes than miscellaneous posts (*P<*.001). On the other hand, posts of recipes and food-related information and posts discussing research and innovation on diabetes received 47% and 31% less likes, respectively, than miscellaneous posts (both *P<*.001). The posts that received fewer comments than miscellaneous posts were those with recipes and food-related information (59% less comments, *P<*.001), posts discussing research and innovation on diabetes (35% less comments, *P=*.02), and posts related to health education (38% less comments, *P=*.003). Health education posts were shared 39% more often than miscellaneous posts (*P=*.007), while posts of recipes and food-related information and posts of research information and innovation on diabetes had 43% and 25% less shares, respectively, than miscellaneous posts (*P=*.02 and *P=*.03, respectively).

With regard to social media channel, Facebook posts were 35 times more likely than Twitter posts to receive likes, 60 times more likely to receive comments, and 13 times more likely to be shared (*P<*.001). Instagram posts were 35 times more likely than Twitter posts to receive likes, and more than 9 times more likely to receive comments (*P<*.001).

In terms of post features, posts that included videos were 3 times more likely to receive likes, almost 4 times more likely to receive comments, and 2.5 times more likely to be shared than text-only posts (all *P<*.001). The addition of both a video and an emoji to a post increased its chances of receiving likes by almost 7 times (*P<*.001), but no effect on comments and shares was observed. Including a picture in the post increased the chances of it receiving likes by 86% and of being shared by 124% (both *P<*.001), but it did not affect the number of comments. By including only an emoji to the text, the chances of posts receiving likes and being shared increased by 71% and 144%, respectively (*P<*.001).

Table 2 shows the negative binomial regression analyses of the effects of content topic, social media channel, and post features as predictors of users’ engagement (likes, comments, and shares).

**Table 2:** Effect of content topic, social media channel, and post features on users’ engagement (likes, comments, and shares).

Independent variables		n	Likes			Comments			Shares		
			OR^a^ (95% CI)	*P* value		OR (95% CI)		*P* value	OR (95% CI)		*P* value
**Content topic**											
	Interviews and personal stories	124	2.11 (1.66-2.68)	*<*.001		2.06 (1.37-3.08)		*<*.001	2.12 (1.58-2.96)		*<*.001
	Awareness days and other celebrations	158	1.41 (1.12-1.76)	.002		1.31 (0.91-1.89)		.15	1.23 (0.88-1.70)		.22
	Recipes and food-related information	99	0.53 (0.40-0.70)	*<*.001		0.41 (0.25-0.65)		*<*.001	0.57 (0.35-0.92)		.02
	Diabetes-related technology	84	0.78 (0.59-1.04)	.09		1.33 (0.83-2.14)		.23	0.73 (0.49-1.06)		.10
	Research and innovation on diabetes	207	0.69 (0.57-0.85)	*<*.001		0.65 (0.45-0.93)		.02	0.75 (0.58-0.97)		.03
	Health education	260	0.87 (0.72-1.04)	.13		0.62 (0.45-0.85)		.003	1.39 (1.09-1.78)		.01
	Miscellaneous	517	1^b^			1^b^			1^b^		
**Social media channel**											
	Facebook	530	35.41 (30.62-40.96)	*<*.001		60.37 (46.77-77.93)		*<*.001	12.79 (10.59-15.45)		*<*.001
	Instagram	201	34.99 (25.13-48.72)	*<*.001		9.68 (5.43-17.28)		*<*.001	N/A^c^		
	Twitter	718	1^b^			1^b^			1^b^		
**Post features**											
	Emoji (no picture, no video)	81	1.71 (1.29-2.26)	*<*.001		1.09 (0.69-1.70)		.70	2.44 (1.69-3.51)		*<*.001
	Emoji and picture	210	1.30 (0.96-1.77)	.09		0.65 (0.37-1.12)		.12	1.33 (0.80-2.19)		.27
	Emoji and video	35	6.83 (4.21-11.09)	*<*.001		1.29 (0.66-2.56)		.45	1.82 (0.91-3.65)		.09
	Picture (no emoji)	342	1.86 (1.58-2.19)	*<*.001		1.12 (0.83-1.49)		.46	2.24 (1.81-2.77)		*<*.001
	Video (no emoji)	79	3.29 (2.26-4.39)	*<*.001		3.87 (2.37-6.31)		*<*.001	2.50 (1.73-3.61)		*<*.001
	Text only (no emoji, no picture, no video)	702	1^b^			1^b^			1^b^		

^a^OR: odds ratio.

^b^Reference group in the corresponding independent variable.

^c^N/A: not applicable.

## Discussion

### Summary

Between January 1, 2017, and December 31, 2019, the Norwegian Diabetes Association posted a total of 1449 messages on their social media channels. The posts that were most engaging to users were those that featured interviews and personal stories. In fact, those posts were twice as likely to receive likes, comments, and shares. On the other hand, posts containing recipes and food-related information, and posts on research and innovation, were the least engaging to social media users. Both types of content were less likely to receive likes, comments, and shares. Regarding the social media channel, Facebook and Instagram posts were both 35 times more likely to receive likes, and 60 times and almost 10 times more likely to receive comments, respectively, than posts on Twitter. Video and emoji were the most engaging post features. Posts with video had 3 times increased chance of receiving likes, almost 4 times increased chance of receiving comments, and 2.5 times increased chance of being shared. Including both video and emoji increased the chances of receiving likes by almost 7 times. The addition of an emoji to a post increased its chances of receiving likes and being shared by 71% and 144%, respectively.

### Content Topic Engagement

We found that diabetes social media posts that engaged the most users were the ones that featured interviews and personal stories, and those that mentioned awareness days and other celebrations. Our results indicate that posting content based on interviews and personal stories on diabetes social media channels offers the highest chances of receiving likes, comments, and shares. This finding contrasts with the results of our previous survey [20], in which interviews and personal stories were the least preferred type of content by diabetes social media users. These findings suggest that there might be a discrepancy between what users say they want and what they actually like. Such a discrepancy might be related to issues such as self-presentation [29], where users prefer to think of themselves as less interested in personal stories than they actually are (and respond accordingly on questionnaires). Researchers have used social media liking patterns to judge users’ personality types, and shown that these judgements are more accurate than those made by users’ close contacts [30]. This could suggest that social media liking patterns more accurately reflect users’ actual interests than do their responses to questionnaires about their preferences.

Posts containing content about awareness days and other celebrations also showed a higher number of likes. Similarly, in a previous study [3] that analyzed 2 diabetes Facebook groups, one open and one closed, posts about awareness days received more likes.

Diabetes social media posts dealing with content that promotes empowerment have been previously linked with higher engagement [25,31]. A study by Harris et al [31] found that tweets that included information about diabetes-related health problems were positively and significantly associated with engagement. Another study [25] reported that posts dealing with diabetes control received more likes, and posts on diabetes’ consequences were associated with greater sharing. In our study, posts that a priori could be linked to greater empowerment, such as those dealing with research and innovation on diabetes or health education, were predictors of less engagement (fewer likes, comments, and shares for research and innovation posts; and fewer comments and shares for health education posts). These results are also in discrepancy with our survey findings [20], in which 78% of respondents indicated that they would like to find more content on research and innovation on diabetes on social media channels. This discrepancy between what users say they want in a survey and what they actually like in real life might also be related to self-presentation issues [29], where users prefer thinking and saying that they are more interested in research than they actually are. Another possible reason for this discrepancy could be that this user group really wants to read about research and innovation but does not feel competent or able to comment or acknowledge by liking and sharing this kind of information.

### Post Features Engagement

The use of videos predicted higher chances of receiving likes, comments, and shares. The inclusion of pictures and emoji also predicted an increased number of likes and shares. Our results are in concordance with previous publications [24,32] that report the use of videos as one of the key features for attracting the greatest amount of user engagement. Technically, videos compel users to stop scrolling for a brief time to perceive and digest the content, which may also be conducive to engagement. Moreover, videos can convey a message in and of themselves and do not need to be accompanied by text, which may increase their effectiveness in communication and their ability to engage users.

Our results on the effect of pictures on engagement are also in line with the findings of a previous study [25] that analyzed 10 diabetes-related Facebook pages, and with a study [24] that focused on engagement with health agencies on Facebook. These two studies [24,25] reported higher rates of liking and sharing of posts with images. Likewise, the use of emoji in social media posts (which are linked to a more positive sentiment) has also been linked to higher levels of user engagement in previous research [32,33].

### Health Implications of Social Media Groups

Disease-specific social media groups, such as the one we analyzed in this study, are recognized as trusted sources of information. Patient associations on social media reach and engage a considerable number of people, which can potentially benefit their users at many levels, including with respect to health outcomes [16,17,19]. These channels could supplement the traditional delivery of information provided by health care professionals today. However, there is still a proportion of people with diabetes who are not benefiting from these channels because they do not use social media channels, they are not interested in belonging to a group discussing their disease (or displaying their health condition by joining the group), or they simply do not know about the existence of such groups.

Analyzing users’ engagement with social media posts is a way for patient associations, healthcare authorities, and other stakeholders to understand the voice of the patient [17] and to know what people are interested in at a specific moment. However, measuring social media engagement as a way of understanding users’ interests could soon face new challenges. Administrators of some popular channels currently believe that showing engagement metrics could be limiting an increase in the volume of posts [34]. In fact, at the end of 2019, Instagram started to hide the number of likes displayed underneath posts in some countries [34]. If these strategies are adopted by more social media channels, alternative approaches to understanding diabetes patients’ interests on these channels would be needed. Alternative strategies to listening to social media users’ interests might include the use of automatic topic classification based on natural language processing or other artificial intelligence techniques, which could help to identify the most popularly discussed or searched themes. The use of sentiment analyses could also help us to understand which of these topics are linked with a more positive or a more negative sentiment.

### Limitations

This study refers to diabetes social media groups led by a national patient association. Although the channels are open, only social media administrators within the patient association are able to post. Users are only able to respond to these posts. The type of content posted by this organization might differ from that posted in other diabetes social media groups.

### Conclusion

Diabetes social media users seem to be least engaged in post content that a priori could be linked to greater empowerment: research and innovation on diabetes, and health education. Diabetes social media groups, public health authorities, and other stakeholders interested in sharing research and innovation content and in promoting health education that engages social media users should consider including videos and emoji in their posts, and preferably publish on popular and visual-based channels, such as Facebook and Instagram, to increase user engagement.

## Abbreviations

API: application programming interface

## References

[1] Stellefson M, Paige S, Apperson A, Spratt S (2019). Social Media Content Analysis of Public Diabetes Facebook Groups. J Diabetes Sci Technol.

[2] Tenderich A, Tenderich B, Barton T, Richards SE (2019). What Are PWDs (People With Diabetes) Doing Online? A Netnographic Analysis. J Diabetes Sci Technol.

[3] Årsand E, Bradway M, Gabarron E (2019). What Are Diabetes Patients Versus Health Care Personnel Discussing on Social Media?. J Diabetes Sci Technol.

[4] Gabarron E, Bradway M, Årsand E (2016). What are diabetes patients discussing on social media?. Int J Integr Care.

[5] Mattson MJ, Hall JG (2011). Linking health communication with social support. Health as communication nexus : a service-learning approach.

[6] Hajek A, König H (2019). The association between use of online social networks sites and perceived social isolation among individuals in the second half of life: results based on a nationally representative sample in Germany. BMC Public Health.

[7] Wong D, Amon KL, Keep M (2019). Desire to Belong Affects Instagram Behavior and Perceived Social Support. Cyberpsychology, Behavior, and Social Networking.

[8] Alzahrani A, Alanzi T (2019). Social Media Use By People With Diabetes In Saudi Arabia: A Survey About Purposes, Benefits And Risks. DMSO.

[9] Kuykendall L, Tay L, Ng V (2015). Leisure engagement and subjective well-being: A meta-analysis. Psychological Bulletin.

[10] Huang C (2017). Time Spent on Social Network Sites and Psychological Well-Being: A Meta-Analysis. Cyberpsychology, Behavior, and Social Networking.

[11] Litchman ML, Edelman LS, Donaldson GW (2018). Effect of Diabetes Online Community Engagement on Health Indicators: Cross-Sectional Study. JMIR Diabetes.

[12] Benetoli A, Chen T, Aslani P (2018). How patients’ use of social media impacts their interactions with healthcare professionals. Patient Education and Counseling.

[13] Househ M, Borycki E, Kushniruk A (2014). Empowering patients through social media: The benefits and challenges. Health Informatics J.

[14] Gavrila V, Garrity A, Hirschfeld E, Edwards B, Lee JM (2019). Peer Support Through a Diabetes Social Media Community. J Diabetes Sci Technol.

[15] Benetoli A, Chen TF, Aslani P (2018). Consumer perceptions of using social media for health purposes: Benefits and drawbacks. Health Informatics J.

[16] Gabarron E, Årsand E, Wynn R (2018). Social Media Use in Interventions for Diabetes: Rapid Evidence-Based Review. J Med Internet Res.

[17] Rozenblum R, Greaves F, Bates DW (2017). The role of social media around patient experience and engagement. BMJ Qual Saf.

[18] Petrovski G, Zivkovic M (2018). Are We Ready to Treat Our Diabetes Patients Using Social Media? Yes, We Are. J Diabetes Sci Technol.

[19] Elnaggar A, Ta Park V, Lee SJ, Bender M, Siegmund LA, Park LG (2020). Patients’ Use of Social Media for Diabetes Self-Care: Systematic Review. J Med Internet Res.

[20] Gabarron E, Dorronzoro E, Bradway M, Rivera-Romero O, Wynn R, Årsand E (2018). Preferences and interests of diabetes social media users regarding a health-promotion intervention. PPA.

[21] Kaur W, Balakrishnan V, Rana O, Sinniah A (2019). Liking, sharing, commenting and reacting on Facebook: User behaviors’ impact on sentiment intensity. Telematics and Informatics.

[22] Wohn DY, Carr CT, Hayes RA (2016). How Affective Is a “Like”?: The Effect of Paralinguistic Digital Affordances on Perceived Social Support. Cyberpsychology, Behavior, and Social Networking.

[23] Greaves F, Ramirez-Cano D, Millett C, Darzi A, Donaldson L (2013). Use of Sentiment Analysis for Capturing Patient Experience From Free-Text Comments Posted Online. J Med Internet Res.

[24] Bhattacharya S, Srinivasan P, Polgreen P (2017). Social media engagement analysis of U.S. Federal health agencies on Facebook. BMC Med Inform Decis Mak.

[25] Rus HM, Cameron LD (2016). Health Communication in Social Media: Message Features Predicting User Engagement on Diabetes-Related Facebook Pages. ann. behav. med.

[26] Gabarron E, Bradway M, Fernandez-Luque L, Chomutare T, Hansen AH, Wynn R, Årsand E (2018). Social media for health promotion in diabetes: study protocol for a participatory public health intervention design. BMC Health Serv Res.

[27] Diabetesforbundet The Norwegian Diabetes Association. Diabetesforbundet.

[28] Landis JR, Koch GG (1977). The Measurement of Observer Agreement for Categorical Data. Biometrics.

[29] Baumeister RD (1987). Self-Presentation Theory: Self-Construction and Audience Pleasing. Theories of Group Behavior.

[30] Youyou W, Kosinski M, Stillwell D (2015). Computer-based personality judgments are more accurate than those made by humans. Proc Natl Acad Sci USA.

[31] Harris JK, Mart A, Moreland-Russell S, Caburnay CA (2015). Diabetes Topics Associated With Engagement on Twitter. Prev. Chronic Dis.

[32] Kite J, Foley BC, Grunseit AC, Freeman B (2016). Please Like Me: Facebook and Public Health Communication. PLoS ONE.

[33] Gabarron E, Dorronzoro E, Rivera-Romero O, Wynn R (2018). Diabetes on Twitter: A Sentiment Analysis. J Diabetes Sci Technol.

[34] Rodriguez S (2019). Facebook has a theory that hiding ‘likes’ will increase post volume, and Instagram is testing that theory. CNBC.

